# Reformulating Psychological Difficulties in People with Parkinson's Disease: The Potential of a Social Relational Approach to Disablism

**DOI:** 10.1155/2013/608562

**Published:** 2013-08-13

**Authors:** Jane Simpson, Helen McMillan, Donna Reeve

**Affiliations:** ^1^Lancaster University, Lancaster LA1 4YT, UK; ^2^Older Adult Psychological Therapies Service, Pennine Care NHS Trust, Ashton-under-Lyne OL6 7SR, UK; ^3^Faculty of Health and Medicine, Lancaster University, Lancaster LA1 4YT, UK

## Abstract

Research investigating the psychological difficulties experienced by people with Parkinson's disease (PD) is dominated by individualistic neurobiological and psychological perspectives. Therefore, this opinion paper draws on a reformulation of the social model of disability, Thomas' (1999) and (2007) social relational approach to disablism, to offer an alternative way of conceptualising psychological difficulties experienced by people with PD. This opinion paper explores the ways in which socially imposed restrictions and stigma may contribute to psychological difficulties by using Thomas' (2007) concept of psychoemotional disablism. By using the lens of psychoemotional disablism, this paper demonstrates that people with PD can be exposed to stigmatising attitudes and interactions which could contribute to restrictions, feelings of shame, and psychological difficulties such as depression. Accordingly, it is argued that further attention to the link between psychological difficulties and social dimensions of disablism in PD is needed in both research arenas and clinical practice to broaden understandings and interventions for people with PD.

## 1. Introduction

In addition to motor symptoms, people with Parkinson's disease (PD) can experience psychological difficulties such as depression [1], anxiety [2], apathy [3], psychosis [4], and problems with impulse control [5] (see [6] for further details). Such nonmotor difficulties can be as, if not more, challenging as motor difficulties to people with PD [7] and their caregivers [8] and are a major contributor to patient perceptions of quality of life (e.g., see [9, 10]). 

In terms of frameworks for understanding the psychological difficulties experienced by people with PD, Brown and Jahanshahi [11] highlighted that neurobiological conceptualisations dominate. Neurobiological conceptualisations assume that psychological difficulties occur as a direct result of pathological neurobiological processes within an individual, such as changes in dopaminergic systems; for example, see [12]. Indeed, this assumption is fundamental in that much psychiatrically driven research on psychological outcomes in people with PD, the primary role given to neurobiological dysfunction is not even questioned, for example, [13, 14]. However, it has been argued that there is a need to broaden conceptualisations of psychological difficulties associated with PD beyond neurobiological models; for example, see [11, 15, 16] and psychological approaches assessing variables such as illness cognitions, personality, and coping styles have developed (see [17–19], e.g.). 

However, psychological models also have their limitations and their critics (e.g., [20, 21]) and even studies including multiple and comprehensive assessments of psychological and clinical predictors [22, 23] cannot fully predict psychological outcomes. It therefore also appears vital to consider the ways in which a person's social, cultural, and economic environment influences their psychological experiences of chronic illness [24]. This is in contrast to individualistic models—from either a neuropsychiatric or a psychological perspective—which assume that disability and psychological difficulties occur due to impairments and emphasise the need for people to seek treatment or adapt to environments dominated by the nonimpaired to appear more “normal” [21, 25]. As such, this paper argues that an alternative approach, a social model perspective of disability, can enhance understanding of psychological difficulties experienced by people with PD, by considering the impact of socially imposed barriers. It is important to stress that this model does not simply describe the finding that social factors can impact psychological outcomes; several studies, even from a psychological or neurobiological perspective, have accepted that social factors (as experienced and measured from an individual perspective) can, albeit in a modest way, influence outcome (e.g., [26]). More radically, it argues that society *actively* disables and limits the potential for well-being of people who display different abilities, and this active disablement is a source of considerable psychological distress. We also argue that in advocating and expounding a causally parsimonious account of dysfunction—where all symptoms, motor or nonmotor, are related to the disease mechanism responsible for the motor dysfunction—researchers are ignoring an important source of distress. 

## 2. A Social Relational Model of Disability

The traditional social model of disability, developed by authors such as Oliver [27], offered a significantly different conceptualisation of disability from the individualistic models of disability [28]. The social model reframed disability as occurring due to societal and environmental barriers driven by nonimpaired values, rather than disability being a direct consequence of physical impairments [27]. Furthermore, as part of a reformulation of the social model of disability, Thomas [24, 29, 30] has produced an extended social relational definition of disablism which explicitly identifies the two dimensions of social oppression that can disable people with impairments (see also Reeve [21, 31, 32]). Firstly, in keeping with the traditional social model, a person with impairment can be disabled by environments that exclude them (e.g., access to facilities and services) which Reeve [33] refers to as *structural disablism. *Secondly, disablement can occur in the interactions between people with and without impairment, impacting a person's self-esteem and psychological well-being, resulting in barriers at an internal, private level [21, 24]. For instance, negative social interactions such as hurtful comments or being stared at can result in a person with an impairment experiencing negative emotions and thoughts about themselves [21]. As such, this can then result in reduced participation in society [21] because of being limited in “who they can be” [31, page 84]. This was referred to as the *psychoemotional dimensions of disability *[24] which has more recently become known as *psychoemotional disablism* [29, 32]. Reeve [32] has further broken down psycho-emotional disablism into two forms. The most significant of these is *direct psycho-emotional disablism* which arises from the relationship that a disabled person has with other people. Therefore, in addition to the impact of negative social interactions described previously, it is also important to take account of *internalised oppression*—in other words, a person internalising negative stereotypes of what it means to have an impairment or illness, resulting in them placing restrictions upon themselves [32]. The second form of psycho-emotional disablism, *indirect psycho-emotional disablism*, is associated with the experience of structural disablism, for example, anger at being excluded from a building or being discriminated against in the work place because of impairment. 

In addition to historical roots based on the experiences of people with physical impairments, social model approaches have been used to enhance the understanding of experiences of people with learning disabilities [34], dementia [35, 36] mental illness [37, 38], motor neuron disease [39], and old age [40]. Despite these perspectives, research has rarely drawn on social model perspectives to understand experiences of PD (an exception being [41] on physical health issues) nor been used to understand the mental health issues experienced by people with PD.

## 3. Indirect Psychoemotional Disablism Arising from Structural Disablism Experienced by People with PD

Structural disablism directly impacts what people can *do* and refers to the disabling barriers which operate at the public level, for example, exclusion from the built environment, discrimination in the work place, or information in inaccessible formats [33, page 24].

Studies that have explored the financial and employment experiences of people with PD provide some insight into the structural barriers that people with PD can face which may contribute to their psychological experience of PD; they are used here as one illustrative example of structural disablism.

PD can result in significant financial costs, such as cost of care provision or moving home [42–45]. It can also affect financial security through forced unemployment due to employers' reluctance to make adaptations to maintain employment opportunities [46]. Although the extent of forced unemployment is not known, one UK study revealed that among people of working age with Parkinson's disease, 46% had stopped working five years after disease onset, and overall more than half had retired before state retirement age [47]. Taking sick leave or finishing work can cause financial implications and stress for people with PD [48, 49] which can contribute to psychological difficulties, particularly as lower socioeconomic status is a known correlate of anxiety and depression [50]. Furthermore, loss of employment may negatively impact a person's level of independence, alter their social role, reduce access to hobbies, and increase social isolation, again, experiences that could contribute to psychological difficulties [47, 51]. Although typically a condition in older adults, PD also affects people under retirement age [52]. The loss of employment can have even more significant financial implications for people with young onset PD and their families in terms of quality of life and psychological difficulties [48, 52].

## 4. Direct Psychoemotional Disablism Experienced by People with PD

Psycho-emotional disablism operates at the private level, restricting people who can *be*. For example, having to deal with hurtful comments, stigmatising actions of others, and internalised oppression which can undermine someone's psycho-emotional well-being and sense of self [33, page 24].

As Reeve [33] suggested, stigmatising interactions between people with and without impairments can result in psycho-emotional disablism [21, 29]. Goffman [53] defined stigma as being a discreditation of a person because they appear to be “different” and have “undesirable” attributes. Link and Phelan [54] extended this conceptualisation and suggested that “stigma exists when elements of labelling, stereotyping, separating, status loss, and discrimination co-occur in a power situation that allows these process to unfold” (page 382). 

In terms of the stigma of PD, numerous quantitative studies using quality of life measures (e.g., Parkinson's Disease Questionnaire [55]) have captured varying levels of self-perceived stigma associated with PD [10, 52, 56–59]. Furthermore, Burgener and Berger [60] found that participants with PD reported similar levels of perceived stigma as a sample of people with Alzheimer's disease (AD). Although the scores were slightly less than the AD sample for the financial insecurity, social rejection, and internalised shame domains, they were slightly higher than the AD sample for social isolation [60]. Furthermore, recent research by Parkinson's UK [61] found that 41% of people with PD report experiencing discrimination because of having PD, including some experiences of misinterpretation of symptoms or verbal abuse in public. This research also found that such incidents frequently occur, with 43% reporting experiencing discrimination or misinterpretation at least once a month [61].

In addition, findings from qualitative studies have captured the nature and impact of stigma associated with PD, and psycho-emotional disablism, for example, “…kids stared at you and you felt like a freak” [62, page 229]. Similarly, a theme in Caap-Ahlgren et al.'s [63] qualitative study of women's experience of living with PD was “perceived stigmatisation” capturing participants' perceptions of negative judgements by people who do not have PD. 

However, research has tended to focus on perceived stigma (i.e., its individual effects) rather than directly investigating socially created exclusion or wider public attitudes about PD [54, 64]. Unfortunately, exceptions to this are few (some published studies are presented in this paper), and it was noted that studies exploring public attitudes are often limited to “grey literature” such as surveys and unpublished research. For example, a European survey of public knowledge of PD found a lack of awareness about the physical symptoms of PD and underestimation of the psychological impact of PD [65]. Similarly, an Australian survey of public attitudes to PD found evidence of some negative attitudes and stereotypes [66]. To understand the nature of psycho-emotional disablism for people with PD, it is important to consider possible reasons why PD is at risk of being stigmatised within public attitudes. For example, Burgener and Berger [60] highlighted that there can be stigma surrounding PD due to movement and communication difficulties. Interestingly, in researching stigma, the focus of investigation is almost always on the person with PD and not on people without PD. 

## 5. The Stigma of Movement Difficulties

Given the nature of some symptoms of PD (e.g., tremors, rigidity) it is plausible that people with PD may be subject to stigmatisation and discredited because of negative societal perceptions of bodily movement [52, 53, 67, 68]. The more visible and less “normal” the symptoms of PD are, the more likely they are to be judged as socially unacceptable or threatening by people who do not have PD [52, 68].

Research findings offer insights into the nature of stigma associated with movement difficulties. For example, Caap-Ahlgren et al. [63] found that women who experienced PD reported discomfort during social interactions because “involuntary movements of arms and legs make them feel especially conspicuous” (page 92). Participants reported that friends and family could be uncomfortable because they lacked understanding of the physical symptoms and so would make comments and ask questions. Such experiences extended to public situations as studies have found that participants with PD experienced other people staring at them [63] or directly expressing irritation at PD symptoms [69]. Furthermore, people with PD movement difficulties can be viewed as less socially desirable [70], and this may manifest as hurtful comments or avoidance of people with PD [71].

In addition, movement difficulties may be misinterpreted by people who are not aware that they are due to PD (e.g., mistaken for being drunk [61, 66]). Furthermore, research has demonstrated that judgments about the unacceptability of movement difficulties may be influenced by the age of the person experiencing PD. Schrag et al. [52] suggested that PD may be viewed as socially unacceptable because it involves a presentation that is synonymous with older age such as slowness of movement, in keeping with Singer's [72] ideas that PD involves premature aging. This is supported by Moore and Knowles [66] who found that younger people viewed PD more negatively than people over the age of 50. Indeed, aging alone can be a source of stigma, with discrimination occurring towards people when they are seen as less competent [73]. 

Additionally, Joachim and Acorn [68] suggested that people may try to conceal having an illness due to fear of stigma. Indeed, some research participants have described trying to hide symptoms of PD by not talking [63] or trying to control body movement [62]. This is considered to be a consequence of psycho-emotional disablism—people feeling they need to conceal impairments to avoid negative interactions and appear more “normal” [21]. The effort involved in this can also have negative psychological consequences. However, “passing” in this manner can have negative psychological consequences; hiding the effects of impairment to pass as “normal” takes physical and emotional effort, and the person is always at risk of exposure if their disability status is suddenly revealed [32]. 

## 6. The Stigma of Communication Difficulties

People who experience PD may be subject to negative actions and stigmatising attitudes about their communication style [60]. For example, there may be misunderstanding and lack of awareness about speech or expressive masking: reduced facial, body, and vocal expressions due to muscular difficulties [74]. People with PD have been perceived by others as being less sociable, less happy, and less friendly due to their speech style [75] and facial masking [76]. Some aspects of personality (e.g., extroversion and neuroticism) of people with PD facial masking are often inaccurately perceived by professionals, particularly by novice professionals [74, 77, 78]. Furthermore, studies have found that carers can often misinterpret how people with PD are feeling [79]. 

Misinterpretation and inaccurate negative judgement about a person's character or feelings may impact on rapport in social interactions and may manifest as incongruent or even negative social interactions with people with PD [74, 78]. This may impact at a psycho-emotional level for the person with PD. However, more experienced professionals were found to be less likely to misinterpret neuroticism than novice professionals, suggesting that experience and awareness of PD contributes to more accurate perceptions of people with PD [77]. Accordingly, raising public awareness about the lesser known features of PD such as facial masking may help reduce negative or misinformed perceptions of people with PD [66] and subsequent psycho-emotional disablism. 

Interestingly, the Jaywant and Pell [75] study explored impressions of people who were unaware that the person they were interacting with had PD. As such, their findings indicated that preconceptions about PD did not influence negative perceptions; instead negative perceptions were formed merely on the basis of speech. As such, public perceptions of people with PD may be negative if their communication is simply considered to be unacceptable [53] rather than being understood to be part of PD. Indeed, Paterson [80] argued that people with speech impairments that perform at a slower pace than what is considered “normal” are marginalised because society values productivity, speed, and efficiency. Furthermore, people with language difficulties may be falsely perceived as incompetent, “senile” [62], or of “mental limitation” [60]. 

Furthermore, local cultural values can influence perceptions of people with PD. Tickle-Degnen et al. [78] found that professionals' perceptions of people with facial masking was influenced by differing Western and East Asian values (e.g., how outgoing or charismatic someone should be). This emphasised the need to consider the influence that cultural attitudes could have on perceptions and misconception of PD, which may fuel stigmatisation [78].

In summary, it seems that public attitudes about PD may be stigmatising due to various visible symptoms of PD being attributed to socially unacceptable characteristics or due to misattribution of PD symptoms as another socially unacceptable behaviour or condition [81]. Worryingly, it appears that some of the lesser known features of PD, such as expressive masking, can result in other people making negative judgements about the character and emotions of people with PD (e.g., see [74]). Furthermore, media representation of PD may contribute to public perceptions of PD. For example, one study [82] found that 8% of American media stories about PD contained stigmatising language or incorrect information.

It is therefore important to consider the impact of this stigmatisation on a person with PD in terms of psycho-emotional disablism. Indeed, stigma associated with a variety of other health conditions has been found to negatively impact a person in many ways, including contributing to emotional stress, anxiety, reduced self-esteem, social isolation, embarrassment, and shame (see [83, 84]).

## 7. The Psychoemotional Impact of Stigma Surrounding PD

The most frequently experienced and researched psychological difficulty in people with PD is depression [85]. Brown and Jahanshahi [11] demonstrated that the pattern of depression varied in a nonlinear way over the course of PD and suggested that depression is not simply a result of increasing impairment. Indeed, Brown and Jahanshahi [11] and Schrag et al. [59] highlighted that “disability” (albeit an individualistic measure of disability) and participants' perceptions of the personal and social impact of PD were stronger predictors of depression than impairment. 

Moreover, studies have found correlations between perceived stigma and depression in people with PD (e.g., [59, 79]). Indeed, repeatedly, participants in qualitative studies have reported embarrassment and shame about having PD [62, 63, 81, 86]. From a social relational perspective, it is argued that these feelings are, in part, a result of stigmatising attitudes and actions of other people because PD is seen as breaking social rules [32, 81]. Indeed, the shame experienced by people with PD can be considered “public shame,” with their home and private world being experienced as safer [81], highlighting the social relational nature of shame for people with PD. 

Furthermore, Reeve [32] argued that psychoemotional disablism can occur at the level of self-identity, therefore, the socially created shame about having PD may be internalised and taken on as part of a person's self-identity. Indeed, some qualitative studies have explored the concept of self identity in PD (e.g., [62, 71]), and participants have described the challenges of living with PD and how it affects their sense of self and their social roles. Charmaz [87] suggested that people with chronic illness experience discreditation of their self identity, which can be influenced by stigmatising and disabling societal views of illness. Whilst Charmaz's views are considered to focus on individualistic aspects of illness experiences; it seems that there is some overlap with aspect of psycho-emotional disablism [29] in that the interactions and views of others can negatively impact a person's sense of identity.

 In addition, Reeve [32] argues that psycho-emotional disablism can also include internalised oppression—a person internalising the negative stereotypes of what it means to have PD and apply these to himself. Reeve [32] suggested that this may be particularly pertinent for people who acquire impairments as adults as they have previously developed perceptions of impairment from a nonimpaired perspective. As such, they may impose their own nonimpaired view of illness such as PD on themselves. An example of this could be experiencing negative feelings about being a burden and striving to sustain independence (e.g., [88]). 

Finally, it is argued that the changes in social contact reported by people with PD could reflect the presence of psycho-emotional disablism. For example, in some studies, participants described choosing to spend time with people who were in a similar position to them rather than people without PD or impairment (e.g., [88]). Other people lacking understanding or being uncomfortable with PD have been cited as a contributory factor to altered social contacts [89]. Indeed, people with PD have reported benefits from spending time with other people with PD in nonstigmatising contexts such as self-help and therapeutic groups (e.g., [90, 91]). Moreover, studies have found that people with PD and their families often avoid social situations due to fear of negative judgement by others [61, 81, 89, 92]. As such, negative and stigmatising public attitudes or action by others are considered to limit social opportunities for people with PD. 

Moreover, it appears that such negative social experiences can contribute to psychological difficulties. For example, social rejection and reduced social contact have been found to be associated with depression in people with PD (e.g., [60, 93]). Furthermore, experiencing psychological difficulties may further fuel the stigma associated with PD, as mental health difficulties in themselves have been found to be stigmatising [37]. Indeed, people with PD have reported being reluctant to take medication or engage in psychological therapy for depression because of the additional stigma of mental health [94].

## 8. Conclusions

By using the lens of psycho-emotional disablism, this paper has demonstrated that people with PD can be exposed to stigmatising attitudes and interactions as they navigate their way through societies that value health and efficiency [80] and devalue difference [53]. Lack of understanding of PD can manifest as misinterpretation of character [77] or negative interactions—stares, avoidance, or questioning of people with PD [63]. As such, this is argued to restrict people with PD and induce feelings of shame and fear [74, 81]. Such feelings can impact a person's self-identity and -esteem and this, in addition to structural barriers, may result in reduced social contact and isolation [89, 93]. Such socially imposed experiences are therefore argued to contribute to the psychological difficulties frequently experienced by people with PD, such as depression and anxiety. 

However, such conclusions are challenging to demonstrate due to research varying in terms of its design, methodology, and quality, making direct comparisons between studies difficult. Furthermore, there is a lack of research directly exploring PD from a social model of disability perspective, and few studies having explored public attitudes about PD, the societal stigma surrounding PD, or how this impacts the mental health of people with PD. As such, it seems imperative that further research is carried out to explore such important issues. 

## 9. Implications for Clinical Practice with People with PD

It is worth acknowledging that the psychological implications of living with PD are widely considered to be under researched but of huge potential importance to the day-to-day management of a complex condition such as PD [95]. Furthermore, it is also agreed, even among those who argue that psychological outcomes such as low mood and anxiety are “neuropsychiatric,” that a range of—individualised—interventions should be developed to help address this [14]. However, in this paper, we argue that change also needs to occur at a societal level with respect to the social barriers faced by people with PD. Furthermore, we argue that, even among those traditionally concerned with promoting and researching individual accounts of the mental health of people with PD, this social level of engagement is vital if we are to improve the mental health of people with PD. There is clear scope for raising public awareness of the varying visible and invisible symptoms to try to alter the stigma associated with PD [66]. Logically, if there are fewer negative perceptions or misconceptions and misattributions about PD, then people with PD should be exposed to fewer negative interactions and less psycho-emotional disablism. However, the efforts needed to effect such a shift would be considerable, and it is perhaps not surprising that seeking to enact change at an individual level though efforts such as medication or psychological therapy offer the prospect of quicker and easier results. Nonetheless, a solely narrow individualistic focus—be it on disordered brains or cognitions—is not sustainable if we are serious about improved mental health.
